# Cardiovascular diseases and guideline-directed medical therapy in low socio-economic status patients: A single center experience

**DOI:** 10.1016/j.jtumed.2026.10.001

**Published:** 2026-10-29

**Authors:** Osama M. Alhadramy

**Affiliations:** Department of Medicine, Faculty of Medicine, Taibah University, Al-Madinah Al-Munawwarah, KSA

**Keywords:** أمراض القلب والأوعية الدموية, عوامل الخطر القلبية الوعائية, العلاج الدوائي الموجّه وفق الإرشادات السريرية, المستوى الاجتماعي والاقتصادي المنخفض, مرض الشريان التاجي, قصور القلب, الرجفان الأذيني, Cardiovascular disease, Low socio-economic status, Risk factor, Therapy

## Abstract

**Objectives:**

Patients with a low socio-economic status (SES) exhibit high cardiovascular risk factors (CVRFs) and a high burden of cardiovascular disease (CVD), but they are under-recognized in clinical practice. In this study, we explored CVRFs and CVD in low-SES patients, and estimated the guideline-directed medical therapy (GDMT) prevalence for each CVD.

**Methods:**

Consecutive individuals with low-SES who attended the primary care and cardiology clinics within a single charitable medical center between 2024 and 2025 were retrospectively recruited. Data on SES, symptoms, CVRFs, CVDs, and treatment strategies were collected and analyzed descriptively. GDMT comprised class I medications reported by patients and documented in their medical records at the time of data collection for each CVD.

**Results:**

Among the 2374 patients, the mean age was 58.7 ± 6 years (57.2% females). Common CVRFs included hypertension (39.5%), diabetes (28.6%), and hyperlipidemia (27.4%). Common CVDs included coronary artery disease (CAD) (12.4%), heart failure (HF) (6%), and atrial fibrillation (AF) (1.7%) (mean ages: 55.79, 63.12, and 62.4 years, respectively). CAD patients had a high prevalence of acute coronary syndrome (74.2%), with most managed noninvasively (87%), but 38.5% received GDMT. HF with reduced ejection fraction (HFrEF) exceeded HF with preserved EF (HFpEF) (63.6% vs. 32.8%). Prevalent HF causes included CAD, hypertension, and valvular disease (33.6%, 27.3%, and 26.6%, respectively). GDMT rates for HFrEF and HFpEF were 18.7% and 34%, respectively. The mean CHA2DS2-VASc score was 3.5 for AF patients, with 42.8% on GDMT (78% on warfarin).

**Conclusions:**

Low-SES patients had a high prevalence of CVRF, where CAD, HF, and AF were most common. The patients were relatively young and managed conservatively, but GDMT rates were low. These findings may guide policymakers to improve cardiovascular care in low-SES individuals.

## الملخص

**الخلفية:**

يُواجه الأفراد ذوو المستوى الاجتماعي والاقتصادي المنخفض عبئاً متزايداً من عوامل خطر الإصابة بأمراض القلب والأوعية الدموية، إلى جانب محدودية الوصول إلى الخدمات الصحية والعلاجات الموصى بها وفق الإرشادات السريرية. وعلى الرغم من أهمية هذه التحديات، لا تزال البيانات المتعلقة بانتشار أمراض القلب والأوعية الدموية ومدى استخدام العلاج الدوائي الموجّه وفق الإرشادات السريرية (GDMT) لدى هذه الفئة في المملكة العربية السعودية محدودة. هدفت هذه الدراسة إلى استكشاف عوامل الخطر القلبية الوعائية والأمراض المرتبطة بها لدى المرضى ذوي المستوى الاجتماعي والاقتصادي المنخفض، وتحديد معدلات استخدام العلاجات الدوائية الموصى بها لكل مرض.

**طرق البحث:**

أُجريت دراسة استعادية وصفية شملت 2374 مريضاً من ذوي المستوى الاجتماعي والاقتصادي المنخفض، ممن راجعوا عيادات الرعاية الصحية الأولية في جمعية الرعاية الصحية الخيرية بالمدينة المنورة، خلال الفترة من يناير 2024 إلى يناير 2025. شملت الدراسة الأفراد البالغين من العمر 18 عاماً فأكثر، ممن كانوا عاطلين عن العمل أو يقل دخل أسرهم الشهري عن 1200 دولار أمريكي. جُمعت البيانات المتعلقة بالخصائص الاجتماعية والاقتصادية، وعوامل الخطر القلبية الوعائية، والأمراض المصاحبة، وأمراض القلب والأوعية الدموية، والعلاجات المستخدمة. وشُخّصت أمراض القلب باستخدام التقييم السريري وتخطيط القلب الكهربائي وتخطيط صدى القلب، مع تقييم استخدام العلاج الدوائي وفق التعريفات المحددة لكل مرض. وحُللت البيانات باستخدام الإحصاء الوصفي، وعُرضت النتائج في صورة تكرارات ونسب مئوية ومتوسطات حسابية وانحرافات معيارية.

**النتائج:**

بلغ متوسط أعمار المشاركين 58.7 ± 6 سنوات، وشكّلت الإناث 57.2% من العينة. وكان معظم المشاركين من غير السعوديين (61.3%)، والعاطلين عن العمل (77.6%)، وغير المشمولين بالتأمين الصحي (68%). وبلغ متوسط الدخل الشهري للأسرة 558 ± 86 دولاراً أمريكياً. وكانت أكثر عوامل الخطر القلبية الوعائية انتشاراً ارتفاع ضغط الدم (39.6%)، وداء السكري (28.6%)، وفرط شحميات الدم (27.4%)، في حين بلغت نسبة المدخنين 6.4%.

كان مرض الشريان التاجي أكثر أمراض القلب والأوعية الدموية شيوعاً (12.4%)، يليه قصور القلب (6%)، ثم الرجفان الأذيني (1.7%). ومن بين 294 مريضاً مصاباً بمرض الشريان التاجي، شُخّص 74.2% بمتلازمة الشريان التاجي الحادة، وخضع 13% فقط لتصوير الشرايين التاجية، بينما تلقى 87% العلاج دون تدخل جراحي أو قسطري. وبلغت نسبة المرضى الذين تلقوا العلاج الدوائي الأمثل وفق الإرشادات 38.5%.

ومن بين 143 مريضاً مصاباً بقصور القلب، شكّل قصور القلب المصحوب بانخفاض الكسر القذفي 63.6%، مقابل 32.8% لقصور القلب المصحوب بكسر قذفي محفوظ. وكانت أبرز مسببات قصور القلب مرض الشريان التاجي (33.6%)، وارتفاع ضغط الدم (27.3%)، وأمراض صمامات القلب (26.6%). وبلغت معدلات استخدام العلاج الدوائي الأمثل 18.7% لدى مرضى قصور القلب ذوي الكسر القذفي المنخفض، و34% لدى المرضى ذوي الكسر القذفي المحفوظ، وفق التعريف الإجرائي المستخدم في الدراسة. كما كانت معدلات استخدام أجهزة مقوّم نظم القلب ومزيل الرجفان القابل للزرع والعلاج بإعادة التزامن القلبي منخفضة، إذ بلغت 7% و7.7% على التوالي.

أما مرضى الرجفان الأذيني، والبالغ عددهم 42 مريضاً، فقد بلغ متوسط أعمارهم 62.4 ± 9.5 سنوات، ومتوسط درجة خطر السكتة الدماغية وفق مقياس CHA_2_DS_2_-VASc نحو 3.5 ± 1.8. وبلغت نسبة المرضى الذين تلقوا العلاج الأمثل بمضادات التخثر 42.8%، وكان الوارفارين أكثر مضادات التخثر استخداماً (78%) مقارنة بمضادات التخثر الفموية المباشرة (22%).

**الاستنتاج:**

أظهرت الدراسة ارتفاع معدلات انتشار عوامل الخطر القلبية الوعائية وأمراض الشريان التاجي وقصور القلب والرجفان الأذيني لدى المرضى ذوي المستوى الاجتماعي والاقتصادي المنخفض. كما كشفت عن انخفاض ملحوظ في معدلات استخدام العلاجات الدوائية الموجّهة وفق الإرشادات السريرية، ومحدودية اللجوء إلى التدخلات القلبية المتقدمة. وتسلط هذه النتائج الضوء على أهمية تحسين إتاحة الرعاية القلبية والعلاجات الموصى بها، وتعزيز برامج الدعم الصحي والدوائي للفئات الأقل دخلاً. ومع ذلك، فإن الطبيعة الاستعادية للدراسة واقتصارها على مركز واحد، وعدم التقييم المنهجي لأسباب عدم استخدام الأدوية، تحدّ من إمكانية تعميم النتائج أو تحديد العوامل المسؤولة عن انخفاض معدلات العلاج.

## Introduction

Socio-economic inequality is a public health challenge for most nations.^1^ In particular, the decline in the incidence of cardiovascular disease (CVD) has been more pronounced among individuals with higher socio-economic status (SES), and the difference in the distributions between higher and lower socio-economic groups is widening.^2^ Moreover, CVD mortality has been increasing in developing countries,^3^ and studies have proposed that addressing socio-economic inequities may reduce the burden imposed by this condition in these countries.^4^

The traditional cardiovascular risk factors (CVRFs) include hypertension, dyslipidemia, diabetes mellitus (DM), family history of premature coronary artery disease (CAD), and smoking. SES measured based on the level of education, income, and occupational status is crucial for the distribution of CVD, and is correlated with adverse cardiovascular outcomes, but it is often overlooked in clinical practice.^5^, ^6^, ^7^

Previous studies found a higher prevalence of CVD in individuals with a low SES than a higher SES.^8^ Groups living in low SES neighborhood levels had higher incidences of CAD, heart failure (HF), and stroke.^9^, ^10^ Importantly, these differences may be due to low-SES individuals exhibiting higher prevalences of hypertension, dyslipidemia, obesity, smoking, and physical inactivity, and they are less likely to receive high-quality health services.^11^, ^12^ Other potential mechanisms underlying this association include cognitive dysfunction, poor nutritional quality, and high stress levels.^13^

Rapid socio-economic development in KSA (a high-income country) has significantly altered the lifestyle of the population;^14^ consequently, the prevalences of CVRFs and CVD exceed those in other high-income countries.^15^ Furthermore, Saudis who live in rural areas have higher rates of DM, obesity, and hypertension compared with those in urban areas.^16^ However, few studies have investigated the effects of SES on the prevalence of CVRFs in low-SES populations in KSA, and epidemiological data on CVDs are also lacking. Studies of these issues may provide insights to help decision makers adapt policies and strategies to mitigate health disparities.

Therefore, in the present study, we aimed to explore CVRFs and CVDs among patients with low SES, and to estimate the prevalence of guideline-directed medical therapy (GDMT) for each CVD.

## Materials and Methods

### Study design, sampling, and setting

This retrospective study included all consecutive low-SES individuals aged 18 years or older who attended the primary care clinics of the Alre'aiah Health Care Society (AHCS) in Al-Madinah Al-Munawwarah, KSA, from January 1, 2024, to January 31, 2025. The AHCS is a non-profit health care organization founded in 2001 that provides free charitable medical services to people with low SES (unemployed individuals or those with a monthly income below $1200). The AHCS is licensed by the Ministry of Health and is under the jurisdiction of the Ministry of Human Resources and Social Development***.*** Eligibility for the charity program was determined through a comprehensive socioeconomic assessment conducted by trained personnel. Each patient underwent a structured interview, and supporting documentation was reviewed to verify their financial status. These documents included salary statements, bank statements, utility bills, rental agreements, and other relevant financial records where available. The $1200 income threshold was based on the verified monthly household income determined through this assessment process. We recruited unemployed individuals and those with a monthly income less than $1200. All patients aged <18 years and those with middle- or high-SES (monthly income > $1200) were excluded.

### Data collection and variables

#### Socio-economic characteristics

Data were collected on the socio-economic characteristics of patients, including their age, sex, nationality, marital status, profession, living arrangement, family size, monthly income, and health insurance status. Educational status was recorded according to the level attained by the participants and categorized into three groups: no education, primary/secondary education, and tertiary education, which included higher secondary school and college. Living arrangements were classified as living alone, with a spouse, or with family members. Family size was categorized into <3, 4–6, and >6 individuals. Health insurance status was identified as uninsured, private, and public insurance provided by the Ministry of Health. Finally, monthly income was classified as < $400, $400–800, and $800–1200.

#### CVRFs and comorbidities

Body mass index (BMI) was calculated based on the weight and height of the patients and categorized as follows: <25, 25–30, 31–35, and >35 kg/m^2^.

Individuals were considered to have DM if they were previously diagnosed, had a fasting glucose level ≥126 mg/dL (7.0 mmol/L), or were under treatment with glucose-lowering therapy. Hypertension was recorded in those with a history of hypertension, current use of antihypertensive treatment, and/or average blood pressure ≥140/90 mmHg. Hyperlipidemia was recorded in those with a previous diagnosis, current use of lipid-lowering therapy, total cholesterol >5.2 mmol/L, and/or low-density lipoprotein cholesterol >3.5 mmol/L. Finally, concomitant comorbidities such as cerebrovascular disease, chronic kidney disease (CKD), chronic liver disease, chronic obstructive pulmonary disease, asthma, anemia (hemoglobin level ≤12 g/dL), and malignancy were recorded.

#### CVDs

Patients with suspected CVD were referred to cardiology clinics and underwent thorough assessment by experienced cardiologists, including electrocardiography (ECG) and echocardiography (ECHO). Participants were diagnosed with CAD if they presented typical anginal pain, which was reported according to the Canadian Cardiovascular Society (CCS) classification, with evidence of ischemic ECG findings and/or the presence of regional wall motion abnormalities on ECHO. Patients with acute coronary syndrome (ACS) were identified as those having anginal pain associated with ischemic changes on ECG and elevated serum troponin levels. Finally, those who underwent coronary angiography were identified and detailed review of their medications was performed. Optimal GDMT for ACS was defined as a combination of (1) dual antiplatelet therapy, (2) statin, (3) beta-blocker, and (4) angiotensin converting enzyme inhibitor/angiotensin receptor blocker (ACEI/ARB) for patients with ejection fraction (EF) less than 40%, hypertension, DM, or CKD according to the current guidelines.^17^

HF was diagnosed according to the Framingham Criteria,^18^ and the dyspnea functional class was reported using the New York Heart Association (NYHA) classification. HF was further categorized according to EF into HF with preserved EF (EF > 50%) (HFpEF), HF with borderline EF (EF = 40–50%) (HFbEF), and HF with reduced EF (EF < 40%) (HFrEF). Furthermore, the etiology of HF was categorized as follows: CAD, regional wall motion abnormalities on ECHO; valvular heart disease (VHD), severe valvular lesion on ECHO; dilated cardiomyopathy (DCM), left ventricle dilated in the absence of other causes of HF; and uncontrolled hypertension, average blood pressure >140/90 mmHg in the absence of other causes. Severe VHD was classified according to the location of mitral regurgitation (MR), mitral stenosis (MS), aortic regurgitation (AR), aortic stenosis (AS), and tricuspid regurgitation (TR). The etiology of VHD was further categorized into rheumatic heart disease (RHD) and non-RHD, which are related to calcification or degeneration. Moreover, RHD was diagnosed according to the Revised Jones Criteria,^19^ and/or based on echocardiographic findings of mitral valve RHD. In addition, optimal GDMT for HFrEF was defined as a combination of: (1) ACEI, ARB, or angiotensin receptor neprilysin inhibitor (ARNI); (2) beta-blocker; (3) mineralocorticoid receptor antagonist (MRA); and (4) sodium glucose-cotransporter 2 inhibitors (SGLT2Is). For this study, GDMT for HFpEF was operationally defined as the use of at least two of the following: (1) ACEI or ARB, (2) SGLT2I, and (3) MRA. SGLT2Is have a Class I recommendation, but Class II recommended therapies were also included to account for the anticipated low use of SGLT2I in our socioeconomically disadvantaged population. Finally, patients with indications for cardiac implantable electronic devices (CIEDs), including implantable cardioverter-defibrillators (ICDs) and cardiac resynchronization therapy (CRT), were identified in the cohort, and the percentages of patients who underwent implantation of these devices were calculated. Participants with indications for CIED and the definition of GDMT for HF were selected according to current guidelines.^20^

Patients were diagnosed with atrial fibrillation (AF) if episodes were detected by ECG. AF was further categorized into valvular AF (in the setting of concomitant moderate-to-severe MS or prosthetic valves) and non-valvular AF. The CHA2DS2-VASc score was calculated for each patient and the mean score was estimated for the cohort. Optimal GDMT for AF was defined as the use of anticoagulation therapy with warfarin or a direct oral anticoagulant (DOAC), as recommended by the guidelines based on the CHA2DS2-VASc score.^21^

Medication data were obtained from the medications reported by patients during the clinic visit and documented in their medical records at the time of data collection.

#### Data analysis

The collected data were imported into the Statistical Package for Social Sciences (SPSS software for Windows, version 22, IBM) for analysis. Descriptive analysis was performed to determine the sample characteristics. Categorical data are presented as numbers and percentages, and data for continuous variables are presented as mean ± standard deviation.

The project adhered to the guidelines of the Declaration of Helsinki and ensured the integrity, privacy, and confidentiality of patient data. This study received ethical approval from our institutional review board (study ID: TU-24-04, dated January 25, 2024).

## Results

### Sociodemographic data

In total, 2374 consecutive patients were enrolled in the cohort (females, 57.2%), with a mean age of 58.7 ± 6 years, where 41%, 37.6%, and 17.7% of the patients were aged 60–79 years, 40–59 years, and more than 80 years, respectively. Most of the patients were married (88.8%), unemployed (77.6%), non-Saudi (61.3%), and living with spouses (98%).

Assessment of the educational levels showed that 50.0% of patients had primary or secondary education, 42.6% had no formal education, and 7.4% had attained tertiary education.

Classification based on family size showed that 53% and 43.9% of the patients had family sizes of >6 and 4–6 individuals, respectively. The mean household monthly income for the cohort was $558 ± ($86), and 13.8%, 77.6%, and 8.6% of the individuals had incomes of < $400, $400–800, and $800–1200, respectively. Analysis of health care insurance coverage showed that 68%, 17.6%, and 14.4% of the cohort were uninsured, had private insurance, and had public insurance, respectively. Table 1 summarizes the socio-economic characteristics of the cohort.

**Table 1:** Socioeconomic characteristics of the cohort (n = 2374).

Characteristic	Frequency and percentage (%)
Age in years	
Mean (standard deviation (SD))	58.7 ± 6
**Age group in years**	
18–39	87 (3.7)
40–59	892 (37.6)
60–79	974 (41)
>80	421 (17.7)
**Sex**	
Male	1017 (42.8)
Female	1357 (57.2)
**Nationality**	
Saudi	918 (38.7)
Non-Saudi	1456 (61.3)
**Marital status**	
Married	2108 (88.8)
Single	266 (11.2)
**Profession**	
Employed	531 (22.4)
Unemployed	1843 (77.6)
**Educational level**	
None	1011 (42.6)
Primary/secondary	1185 (50)
Higher school	146 (6)
College	32 (1.4)
**Living arrangement**	
Alone	46 (2)
With spouse/another person	2328 (98)
**Family size**	
≤3	75 (3.1)
4–6	1043 (43.9)
≥6	1256 (53)
**Monthly income ($)**	
Mean (SD)	558 (86)
<400	327 (13.8)
400–800	1843 (77.6)
800–1200	204 (8.6)
**Insurance status**	
Public	342 (14.4)
Private	418 (17.6)
Uninsured	1614 (68)

The mean BMI for the patients was 28.05 ± 4.4 kg/m^2^. The majority of individuals had a BMI <25 (68%), and 32% were either overweight or obese. Analysis of CVRFs showed that 5.3% of the individuals had no risk factors, and the most common CVRFs were hypertension (39.6%), DM (28.6%), hyperlipidemia (27.4%), and cigarette smoking (6.4%). Moreover, the prevalent comorbidities were CKD (18.6%), asthma (12.3%), anemia (9.7%), and cerebrovascular disease (5.7%). Finally, CAD (12.4%), HF (6%), and AF (1.7%) accounted for the majority of CVD cases. The clinical characteristics of the patients are summarized in Table 2.

**Table 2:** Clinical characteristics of the cohort (n = 2374).

Characteristic	Frequency and percentage (%)
**Anthropometric data**	
BMI, mean (standard deviation)	28.05 ± 4.4
<25	1614 (68)
25–30	380 (16)
31–35	224 (9.4)
>35	156 (6.6)
**Cardiovascular risk factors**	
No risk factors	126 (5.3)
Current smoker	151 (6.4)
Hypertension	940 (39.6)
Diabetes	681 (28.6)
Hyperlipidemia	553 (27.4)
**Comorbidities**	
Peripheral artery disease	76 (3.2)
Cerebrovascular disease	136 (5.7)
Chronic kidney disease	442 (18.6)
Chronic liver disease	43 (1.8)
COPD	37 (1.5)
Asthma	294 (12.3)
Anemia (hemoglobin ≤12 g/dL)	232 (9.7)
Malignancy	28 (1.2)
**Cardiovascular diseases**	
CAD	294 (12.4)
HF	143 (6)
AF	42 (1.7)

BMI: body mass index; COPD: chronic obstructive pulmonary disease; CAD: coronary artery disease; HF: heart failure; AF: atrial fibrillation.

### CAD

Table 3 summarizes the findings for 294 patients (12.4%) diagnosed with CAD. Their mean age was 55.79 ± 13.03 years (males, 67.4%). The most frequent CVRFs were hyperlipidemia (63.9%), DM (38.4%), cigarette smoking (36.4%), and hypertension (28.6%). Analysis of angina classes showed that 63.3% and 36.7% of the patients reported angina in CCS classes III and I–II, respectively. Furthermore, ACS was diagnosed in 74.2% of the patients, where 13% and 87% of them underwent coronary angiography and were managed noninvasively, respectively. Assessment of medical therapy showed that 92.2% of patients were receiving aspirin, 10.6% clopidogrel, 4.1% ticagrelor, 87.7% beta-blockers, 86.4% nitrates, 6.1% ACEIs/ARBs, and 23.4% statins.

**Table 3:** Characteristics and treatment of patients with coronary artery disease (n = 294).

Characteristic	Frequency and percentage (%)
Age, mean (standard deviation)	55.79 ± 13.03
Males	198 (67.4)
Hypertension	84 (28.6)
DM	113 (38.4)
Hyperlipidemia	188 (63.9)
Current smoker	107 (36.4)
ACS	218 (74.2)
**Angina class**	
CCS I–II	108 (36.7)
CCS III–IV	186 (63.3)
Management	
Coronary angiogram	38 (13)
Noninvasively	244 (87)
**Medications**	
Aspirin	271 (92.2)
Clopidogrel	31 (10.6)
Ticagrelor	12 (4.1)
Beta-blocker	258 (87.7)
Nitrate	254 (86.4)
Statins	69 (23.4)
ACEI/ARB	18 (6.1)
GDMT for ACS	113 (38.5)

DM: diabetes mellitus; ACS: acute coronary syndrome; CCS: Canadian Cardiovascular Society; ACEI: angiotensin converting enzyme inhibitor; ARB: angiotensin receptor blocker; GDMT: guideline directed medical therapy.

Optimal GDMT was observed in 38.5% of the patients with ACS.

### HF

Table 4 summarizes the findings for 143 patients (6%) diagnosed with HF, where 32.8%, 3.5%, and 63.6% had HFpEF, HFbEF, and HFrEF, respectively. Their mean age was 63.12 ± 11.5 years (females, 58.7%). The most frequent CVRFs were hypertension (65%), DM (49.6%), hyperlipidemia (23%), and cigarette smoking (11.2%). At presentation, 60.8% and 39.2% of the patients exhibited dyspnea in NYHA class III–IV and NYHA class I–II, respectively. The most common causes of HF were CAD (33.6%), hypertension (27.3%), VHD (26.6%), and DCM (12.5%). In addition, the majority of VHDs were MR (44.7%), MS (26.3%), TR (23.7%), AS (18.4%), and AR (10.5%), with most related to RHD (60.5%).

**Table 4:** Characteristics and treatments of patients with heart failure (n = 143).

Characteristics	Frequency and percentage (%)
Age, mean (standard deviation)	63.12 ± 11.5
Females	84 (58.7)
Hypertension	93 (65)
DM	71 (49.6)
Hyperlipidemia	33 (23)
Current smoker	16 (11.2)
**Classification of HF**	
HFpEF	47 (32.8)
HFbEF	5 (3.5)
HFrEF	91 (63.6)
**Dyspnea NYHA class**	
NYHA I–II	56 (39.2)
NYHA III–IV	87 (60.8)
**Etiology of heart failure**	
Valvular heart disease	38 (26.6)
RHD	23 (60.5)
Non-RHD	15 (39.5)
MR	17 (44.7)
MS	10 (26.3)
AR	4 (10.5)
AS	7 (18.4)
TR	9 (23.7)
DCM	18 (12.5)
CAD	48 (33.6)
Hypertension	39 (27.3)
**Medications**	
ARNI	11 (7.7)
ACEI/ARB	56 (39.2)
MRA	18 (12.6)
Diuretic	124 (86.7)
Digitalis	6 (4.2)
Beta-blocker	48 (33.6)
SGLT2I	21 (14.7)
GDMT for HFrEF	17 (18.7)
GDMT for HFpEF	16 (34)
**CIED**	
ICD	4/58 (7)
CRT	2/26 (7.7)

DM: diabetes mellitus; HF: heart failure; HFpEF: heart failure with preserved ejection fraction; HFbEF: heart failure with borderline ejection fraction; HFrEF: heart failure with reduced ejection fraction; NYHA: New York Heart Association; RHD: rheumatic heart disease; MR: mitral regurgitation; MS: mitral stenosis; AR: aortic regurgitation; AS: aortic stenosis; TR: tricuspid regurgitation; DCM: dilated cardiomyopathy; CAD: coronary artery disease; ARNI: angiotensin receptor neprilysin inhibitor; ACEI: angiotensin converting enzyme inhibitor; ARB: angiotensin receptor blocker; MRA: mineralocorticoid receptor antagonist; SGLT2I: sodium-glucose cotransporter 2 inhibitor; GDMT: guideline directed medical therapy; CIEDs: cardiac implantable electronic devices; ICD: implantable cardioverter-defibrillator; CRT: cardiac resynchronization therapy.

Analysis of medical therapy showed that 7.7% of patients were receiving ARNIs, 39.2% ACEIs/ARBs, 12.6% MRAs, 86.7% diuretics, 4.2% digitalis, 33.6% beta-blockers, and 14.7% SGLT2Is. Optimal GDMT was observed in 18.7% and 34% of patients with HFrEF and HFpEF, respectively. Among reported CIEDs, 4/58 (7%) and 2/26 (7.7%) patients received ICD and CRT, respectively.

### AF

Table 5 summarizes the findings for 42 patients (1.7%) diagnosed with AF (females, 69%). Overall, 81% and 19% of the patients exhibited nonvalvular AF and valvular AF, respectively. Their mean age was 62.4 ± 9.5 years, with a mean CHA2DS2-VASc score of 3.5 ± 1.8. The most common CVRFs were hypertension (73.8%), DM (66.7%), hyperlipidemia (38.1%), and cigarette smoking (7.1%).

**Table 5:** Characteristics and treatments of patients with atrial fibrillation (n = 42).

Characteristic	Frequency and percentage (%)
Age, mean (standard deviation (SD))	62.4 ± 9.5
CHA2DS2-VASc score, mean (SD)	3.5 (1.8)
Females	29 (69)
Hypertension	31 (73.8)
DM	28 (66.7)
Hyperlipidemia	16 (38.1)
Current smoker	3 (7.1)
**Type of AF**	
Non-valvular	34 (81)
Valvular	8 (19)
**Medications**	
DOAC	4 (22)
Warfarin	14 (78)
GDMT	18 (42.8)

DM: diabetes mellitus; AF: atrial fibrillation; DOAC: direct oral anticoagulant; GDMT: guideline directed medical therapy.

Analysis anticoagulation usage showed that 22% and 78% of patients were on DOAC and warfarin, respectively, but optimal GDMT was only observed in 42.8% of the patients.

## Discussion

This study explored CVD and its risk factors among 2374 individuals with low SES, as evidenced by low monthly income, low level of education, and high unemployment rate. We identified a high prevalence of risk factors, where CAD, HF, and AF were the most common CVDs. These patients were relatively young and had a high cardiovascular risk profile, and most did not receive GDMT according to the current guidelines.

### CVRFs

The prevalence of hypertension in the current study was 39.6%, which is higher than national levels (15–30%),^22^ and that among low SES populations in high-income countries (28%).^23^ Lower SES is consistently associated with a higher prevalence of hypertension because of the higher cost of healthier diets and increased consumption of fast and processed foods.^24^

The prevalence of obesity and overweight was 32%, which is comparable to the national level,^25^ but higher than those reported globally among low SES populations (10–22%).^26^ In KSA, low education level and income are associated with a higher prevalence of obesity.^25^ It has been reported that low-SES groups consume more carbohydrate-rich meals compared with those in high SES groups.^24^ In addition, higher levels of psychological distress and subsequent emotional eating among individuals with low SES have been linked to obesity.^27^

DM was diagnosed in 28.6% of the patients, which is slightly higher than the national level (23%),^28^ and double the rates found among low-SES patients in high-income countries.^29^ A previous study reported that people in the lowest SES group had a 40% higher risk of DM compared with those in the highest SES group.^30^ Furthermore, low SES is associated with lower levels of physical activity, poor diet quality, and consumption of foods that are high in calories, carbohydrates, and fat, which are cheaper than foods high in fiber and vegetables, and they contribute significantly to the development of type 2 DM.^31^

Hyperlipidemia was diagnosed in 27.4% of the patients, which is similar to the national prevalence (30%),^32^ but higher than the global rate (18%).^33^ Unhealthy lifestyles among individuals with low SES, such as physical inactivity, sedentary lifestyles, and consumption of fatty foods, are important risk factors for obesity.^34^

### CVDs

The prevalence of CAD was 12.4%, which is twice the estimated national value (5.5%).^35^ The mean age of the patients was 55.79 years, which is comparable to that in other studies conducted in low-income countries.^36^ However, patients were a decade younger and had a higher CVRF than those with CAD from low SES groups in high-income countries.^37^ Low SES has been associated with premature CAD, which is attributed to a high prevalence of CVRFs, decreased access to nutrition and a healthier lifestyle, and low levels of physical activity.^38^

At diagnosis, most patients with CAD (63.3%) presented with advanced functional angina class (CCS class III–IV), and 74.2% were diagnosed with ACS based on symptoms, ECG, and elevated serum troponin. These findings are consistent with previous research; in particular, compared with high SES patients, patients with low SES exhibit more severe clinical presentations and a higher incidence of ACS, complications, and mortality.^39^ Analysis of the management of patients with ACS showed that the majority were treated noninvasively (87%), and a coronary angiogram was performed for 13% of the patients. Most patients in the cohort were uninsured (68%), but the low rate of coronary angiography cannot be attributed only to a lack of health insurance because irrespective of insurance coverage, the majority of patients with ACS in KSA receive complete emergency medical care, including coronary angiography, as a life-saving procedure without the need for advance payment. The low rate of invasive treatment appeared to be primarily related to limited referral for invasive evaluation and financial barriers as many patients were concerned about the costs of procedures. In high-income countries, only 8–14% of low-income patients with ACS are referred for cardiac catheterization.^40^

In the present study, the rate of GDMT for ACS was low (38.5%), but we found high prescription rates for aspirin (92.2%) and beta-blockers (87.7%). Among GDMT for ACS, aspirin and beta-blockers are the cheapest and most affordable, potentially explaining this disparity. A recent meta-analysis showed that the prescription rate of GDMT for ACS was low (40–60%) among low SES groups, except for aspirin.^41^

The mean age of patients with HF was 63 years, which is 10 years younger than that for those with HF in high-income countries,^42^ and similar to that for those in low-income countries.^43^ They also had high prevalences of CVRFs, CAD, and RHD, and 60% exhibited advanced functional symptoms (NYHA III–IV) at the time of diagnosis. Among low SES groups, 50–65% of patients with newly diagnosed HF presented late and had more severe symptoms than those with higher SES.^44^ The most common causes of HF in this cohort were CAD, hypertension, VHD, and DCM, which are similar to the leading causes of HF in high-income countries.^45^ Among these patients, the prescription rates for HF medications were high for diuretics, modest for beta-blockers and ACEIs/ARBs, and extremely low for ARNI and SGLT2Is. However, the rates of optimal GDMT for HFrEF (18.7%) and HFpEF (34%) were lower than those reported among low-SES HF patients in high-income countries and similar to the rates observed in low-income countries.^46^, ^47^ Furthermore, the utilization of CIEDs was very low for ICD (7%) and CRT (7.7%), and even lower than the rates reported in some low-income countries.^48^ Based on our clinical experience regarding the low rate of device therapy, this was probably attributable to limited referral for device evaluation and financial barriers.

Patients with AF exhibited an increased burden of comorbidities and an elevated mean CHA2DS2-VASc score, but the rate of optimal GDMT (i.e., anticoagulation therapy), was only 42.8%, indicating that it was not received by more than half of the eligible patients. Low SES has been linked to both an increased risk of AF and low anticoagulant use compared with high-SES groups.^49^ The anticoagulant used by most patients was warfarin (78%), and DOACs were prescribed to 22% of them. Among patients with AF, low SES has consistently been associated with lower use of DOAC and higher use of warfarin; however, the DOAC usage rate in the current study was lower than that reported in high-income countries.^50^

The findings obtained in the present study should be carefully interpreted in the context of the following limitations: (1) the retrospective nature of the study limited the ability to attribute causation; (2) the results should not be generalized as they were derived from a single center and a small sample size of patients with CVD; (3) the majority of the cohort were non-Saudis and did not have healthcare coverage, which could have influenced the outcomes; and (4) prescription data, medication adherence, and reasons for non-use were not systematically collected, and thus we could not distinguish whether the observed low GDMT rates were attributable to physician prescribing practices, medication affordability, or other barriers. However, the findings highlight a substantial gap between guideline recommendations and reported medication use, demonstrating the need for both the improved implementation of GDMT by healthcare providers and healthcare policies that address financial barriers among socioeconomically disadvantaged populations.

Nonetheless, the study had several strengths, including a well-characterized and diverse cohort by age, sex, and race. We also determined detailed cardiovascular data and the management strategies for patients, and compared them with the recommended GDMT.

This study highlights multiple potential barriers that may limit access to cardiovascular care among socioeconomically disadvantaged patients, including financial constraints, limited healthcare access, under-referral to specialist services, medication costs, and low health literacy. Potential solutions include expanding charitable medication assistance programs, encouraging donation, promoting corporate social responsibility initiatives, strengthening public–private partnerships, and implementing policies to improve medication affordability.

## Conclusion

In this single-center study in KSA (a high-income country), individuals with low SES had a high prevalence of CV risk factors, where CAD, HF, and AF were the most common. At the time of diagnosis, these patients were relatively young. The majority presented with more severe and advanced symptoms, and tended to have a high CV risk profile. Analysis of treatment strategies showed that most individuals were managed conservatively, and few underwent invasive procedures (catheterization and CIED), and the rate of GDMT was low.

## Ethical approval

This project adhered to the guidelines of the Declaration of Helsinki and the integrity, privacy, and confidentiality of patient data were ensured. This study received ethical approval from our institutional review board (study ID: TU-24-04, dated January 25, 2024).

## Authors contributions

The author is responsible for conceptualization of the study, methodology, data collection, and writing the manuscript.

## Source of funding

This research did not receive any specific grant from funding agencies in the public, commercial, or not-for-profit sectors.

## Conflict of interest

The authors have no conflict of interest to declare.
